# Federated Learning-Based Model for Predicting Mortality: Systematic Review and Meta-Analysis

**DOI:** 10.2196/65708

**Published:** 2025-07-21

**Authors:** Nurfaidah Tahir, Chau-Ren Jung, Shin-Da Lee, Nur Azizah, Wen-Chao Ho, Tsai-Chung Li

**Affiliations:** 1 Department of Public Health, College of Public Health, China Medical University, No. 100, Section 1, Jingmao Road, Beitun District, Taichung, 406040, Taiwan, 886 422053366 ext 6117; 2 Department of Industrial Engineering, Hasanuddin University, Makassar, Indonesia; 3 Japan Environment and Children’s Study Programme Office, National Institute for Environmental Studies, Tsukuba, Japan; 4 Department of Physical Therapy, Healthcare Science Program, China Medical University, Taichung, Taiwan

**Keywords:** federated learning, centralized machine learning, mortality prediction

## Abstract

**Background:**

The rise of federated learning (FL) as a novel privacy-preserving technology offers the potential to create models collaboratively in a decentralized manner to address confidentiality issues, particularly regarding data privacy. However, there is a scarcity of clear and comprehensive evidence that compares the performance of FL with that of the established centralized machine learning (CML) in the clinical domain.

**Objective:**

This study aimed to review the performance comparisons of FL-based and CML models for mortality prediction in clinical settings.

**Methods:**

Experimental studies comparing the performance of FL and CML in predicting mortality were selected. Articles were excluded if they did not compare FL with CML or only compared the effectiveness of different FL baseline models. Two independent reviewers performed the screening, data extraction, and risk of bias assessment. The IEEE Xplore, PubMed, ScienceDirect, and Web of Science databases were searched for articles published up to June 2024. The risk of bias was assessed using CHARMS (Checklist for Critical Appraisal and Data Extraction for Systematic Reviews of Prediction Modeling Studies) and PROBAST (Prediction Model Risk of Bias Assessment Tool). Meta-analyses of the pooled area under the receiver operating curve (AUROC)/area under the curve (AUC) were performed for within-group comparisons (before and after federation).

**Results:**

Nine articles with heterogeneous framework design, scenario, and clinical context were included: 4 articles focused on specific case types; 3 articles were conducted in intensive care unit settings; and 2 articles in emergency departments, urgent centers, or trauma centers. Cohort datasets involving 1,412,973 participants were used in all of the included studies. These studies universally indicated that the predictive performance of FL models is comparable to that of CML. The pooled AUC for the FL and CML performances were 0.81 (95% CI 0.76‐0.85; *I*^2^=78.36%) and 0.82 (95% CI 0.77‐0.86; *I*^2^=72.33%), respectively. The Higgins *I*^2^ test indicated high heterogeneity between the included studies (*I*^2^≥50%). In total, 4 out of 9 (44%) of the developed models were identified as having a high risk of bias.

**Conclusions:**

This systematic review and meta-analysis demonstrate that FL can achieve similar performance to CML while conquering privacy risks in predicting mortality across various settings. Owing to the small number of studies and a moderate proportion of the high risk of bias, the effect estimates might be imprecise.

## Introduction

Predicting mortality is essential in medicine, and numerous tools have been developed for clinical settings. The accurate prediction of mortality enables health care providers to manage treatment planning and resource allocation [1]. Estimating the likelihood of mortality at the end of an intensive care unit (ICU) stay or within a designated timeframe is an effective means of prioritizing care by optimizing staff and equipment use. In addition, such a prediction model improves personalized treatment, especially for individuals facing terminal illness, by identifying patients who may benefit from receiving palliative care, allowing the care plans to align with patient prognoses and preferences [2].

The emergence of machine learning (ML), as a subset of artificial intelligence, has contributed to the development of computational thinking. ML empowers computers to “learn” from training data and augment their knowledge without the need for explicit programming. ML algorithms can identify patterns from data and use this knowledge to generate predictions. Thus, ML models and algorithms can acquire knowledge based on experience. Integrating ML models to assess mortality risk in clinical workflows enables real-time monitoring, which allows physicians to stratify patients according to their severity and rapidly respond to changes in patient states [3]. Despite the potential benefits of ML models in tailoring clinical interventions, hospitals typically have limited local data available to create reliable models [4].

Sharing additional datasets from various health care facilities can significantly enhance the performance and generalizability of ML models [5]. This underscores the critical role of data sharing in the advancement of high-performance predictive models in clinical environments. However, within the health care sector, it is common for hospitals to isolate their datasets, often justifying this practice with legitimate privacy concerns while developing an internal model [6]. Despite the belief of hospitals in the benefits of data sharing, conducting analysis in a centralized manner, which necessitates the consolidation of datasets from all participating hospitals or centers, heightens the risks associated with data privacy and security, as sensitive information is now disseminated to external entities. Furthermore, the transfer of datasets to a centralized repository, whether through physical means or network channels, creates additional vulnerability for potential data breaches [7,8]. In addition to privacy and security challenges, the administrative burden of orchestrating data sharing is significant, as each participant typically adheres to its own regulations concerning data use and ownership [8]. Consequently, a methodology that facilitates collaborative modeling in a decentralized framework, eliminating the requirement to aggregate all datasets in a single location, would significantly enhance the feasibility of multicenter studies.

Federated learning (FL) has emerged as a novel privacy-preserving technology that offers the potential to create models collaboratively in a decentralized manner to address confidentiality issues, particularly in terms of data privacy. FL was introduced by Google in 2016 [9]. The architecture of FL aims to eliminate data exchange between participants. As a collaboratively distributed or decentralized ML privacy-preserving technology, FL eliminates the need to transfer data from the nodes to a central server. The principle of FL, or client-based architecture, enables multiple institutions to collaborate, wherein the baseline model is hosted by a coordinating node and computational nodes download the model and train it on local datasets. FL attempts to formulate models from various datasets and merges knowledge into a globally trained model, which increases the model’s efficiency. Offering viable solutions for investigating medical conditions [10], particularly those with scarce prevalence or minimal data, to prevent inadequate care resulting from misrepresentation or underrepresentation of certain patient groups [11].

Despite its numerous benefits, FL has not yet been extensively implemented in clinical settings, and initiatives aimed at enhancing clinical translation are currently in progress. In addition, as a new emerging technology focused on privacy preservation, investigations into the robustness of FL within multiple clinical fields, along with comparative studies against established ML methodologies, are ongoing. In recent years, only a few studies have reviewed the potential benefits of FL in the clinical environment [4,12,13]; to the best of our knowledge, comparisons between FL and centralized machine learning (CML) approaches in performing clinical tasks have not yet been quantitatively assessed. Thus, we performed a systematic review and meta-analysis to examine the performance of the FL approach in comparison with single-center–based CML in predicting mortality, evaluate the barriers to widespread clinical adoption, and provide insights into future directions of FL-related research in the health care domain. In this context, this study attempts to answer the following question: “What is the feasibility and capability of an FL approach for predicting mortality compared with a CML-based model?”

## Methods

### Design

This systematic review and meta-analysis were conducted in accordance with the PRISMA (Preferred Reporting Items for Systematic Reviews and Meta-Analyses) guidance [14] (Checklist 1).

### Eligibility Criteria

Articles published after 2016 were eligible for inclusion if they investigated research evidence in a clinical context. Articles were included if they quantitatively compared FL and CML models in predicting mortality (before and after federated within-group comparisons) in terms of area under the receiver operating curve (AUROC)/area under the curve (AUC). The eligible study designs included experimental studies that compared the performances of FL and CML. The outcome of interest was mortality prediction.

Articles were excluded if they did not compare FL with CML or only compared the effectiveness of FL performance in different FL baseline models. The excluded studies consisted of protocols, reviews, studies using only qualitative methods, opinion pieces, and conference abstracts without linked full-text articles. Articles were also excluded if they evaluated the model performance with evaluation metrics other than the AUROC/AUC and if they were not available in English.

### Information Sources

A search was conducted in 4 multidisciplinary databases (IEEE Xplore, PubMed, Science Direct, and Web of Science) using EndNote 20 software (Clarivate). The date of the last search was June 23, 2024. Manual searches of the reference lists, citations, and related articles of the included studies were performed to identify additional studies that were missed in the original electronic searches.

### Search Strategy

The controlled free-text terms were used through Boolean operators (Multimedia Appendix 1). All original studies that developed an ML mortality prediction model were included if they met the predefined inclusion criteria:

- Population: Patients in different clinical settings (eg, ICU, emergency department [ED], trauma centers, or specific disease admission).
- Intervention: FL model.
- Comparator: CML model.
- Outcomes: Mortality prediction.

### Selection Process

Records from the electronic and citation searches were exported to EndNote Online for deduplication, followed by title, abstract, and full-text screening. One reviewer (NT) extracted the data from all identified studies using a predefined data extraction form, and a second reviewer (NA) checked the data for accuracy. Any disagreements between the reviewers were resolved by consensus. If neither reviewer reached a consensus, a third reviewer (the corresponding author) made the final decision.

### Data Collection

Data from the included studies were independently extracted by 2 reviewers (see Selection Process section) using a data extraction form developed a priori. The accuracy of data extraction was confirmed by comparing the extraction forms and returning them to the original article to resolve any disparities.

### Data Items

The variables collected were study characteristics, including the data source, number and description of participants, predictors, model development approach, and model performance comparison. For the outcome of interest, the AUROC/AUC, variance, and sample sizes were extracted for each comparison. When these data were missing, they were calculated from other reported statistics using recommended methods [15] where possible. For studies that reported multiple outcome measures, only the outcome of interest (mortality prediction) was collected.

### Risk of Bias Assessment

Two reviewers independently assessed the risk of bias using the PROBAST (Prediction Model Risk of Bias Assessment Tool) [16]. Disagreements were resolved through discussion with other researchers. The PROBAST includes 20 signaling questions across 4 key domains (participants, predictors, outcome, and analysis), and each domain is assessed for a low, high, or unclear risk of bias. The CHARMS (Checklist for Critical Appraisal and Data Extraction for Systematic Reviews of Prediction Modeling Studies) was also examined in conjunction with the PROBAST tool [17]. The PROBAST tool, its considerations, and related publications are available on the PROBAST website [18]. Multimedia Appendix 2 provides a summary of the criteria used to determine the risk of bias.

### Data Synthesis

The included studies were summarized narratively in text, tables, and figures. The discriminant ability, namely the capability to distinguish surviving patients and death events, of the prediction model was extracted (ranging from 0.5 [no discriminative ability] to 1 [perfect discriminative ability]) [15]. Owing to the lack of calibration plots and summaries of calibration, the agreement between the frequency of observed events and the predicted probabilities was not assessed. Prognostic prediction models with effect sizes (AUROCs) for the same outcome were synthesized and analyzed using the metafor package in R (version 4.3.3; R Core Team, R Foundation for Statistical Computing). As the included studies typically differ in design and execution (Multimedia Appendix 3 [19-27]), variations in their results are unlikely to occur by chance only. Thus, standard errors were estimated based on a normal distribution assumption. In addition, the presence of heterogeneity was considered, and the summary result with its 95% CI, which quantified the average performance across studies, was assessed by implementing a random, rather than fixed-effect, meta-analysis model [14,15]. We evaluated the heterogeneity between the included studies using the Higgins *I*^2^ test (*I*^2^≤25% for low, *I*^2^<50% for moderate, and *I*^2^≥50% for high) (Multimedia Appendix 4 [24]).

### Certainty Assessment

The performance of FL and CML for each outcome was evaluated using the C statistic. When measures of uncertainty were not reported, we approximated the standard error of the C statistic using the appropriate and suggested measurements (Multimedia Appendix 4 [24]).

## Results

**Figure 1.**
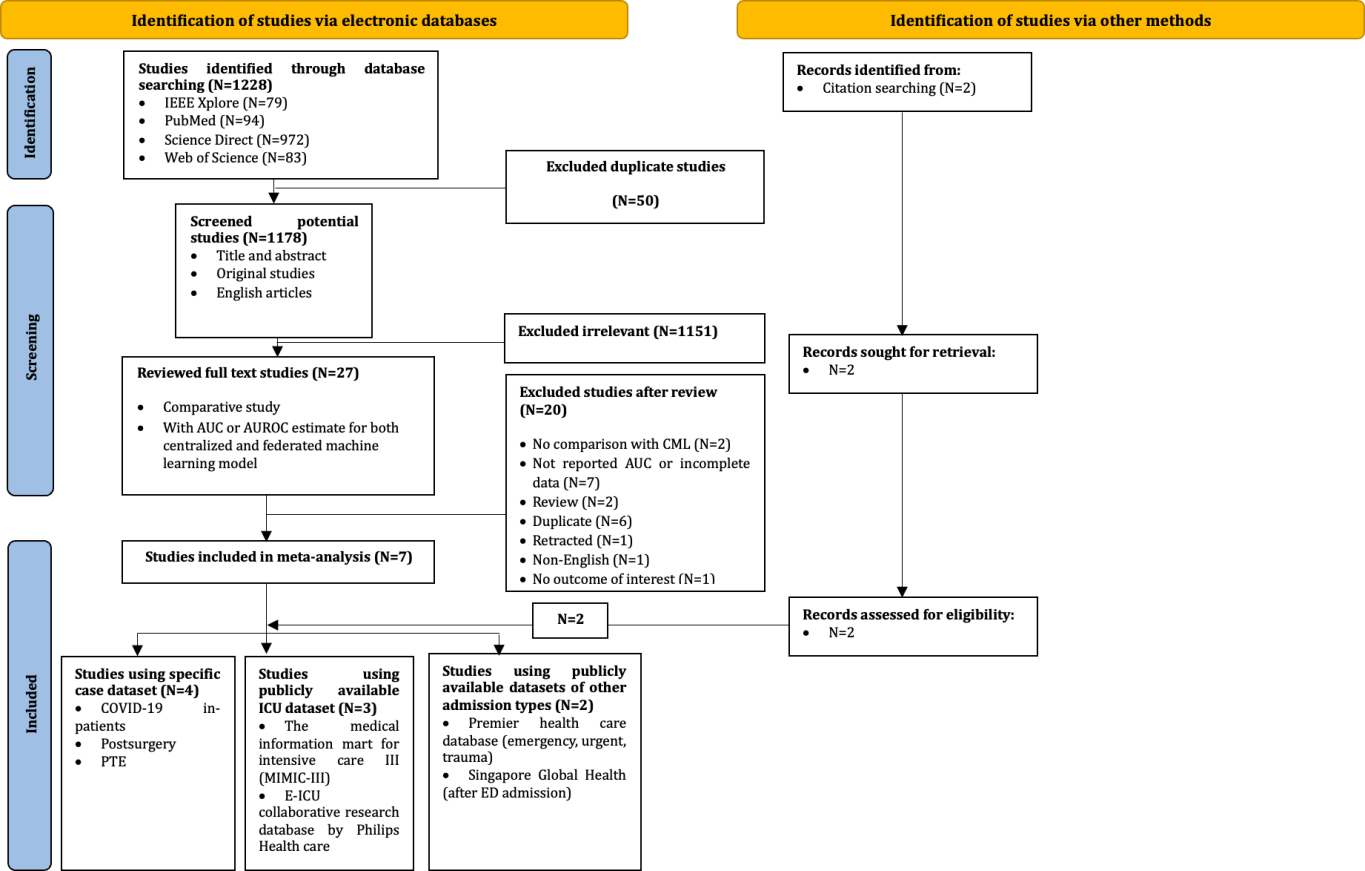
PRISMA (Preferred Reporting Items for Systematic Reviews and Meta-Analyses) flow diagram illustrating the process of the study selection for a systematic review on the federated learning–based model for predicting mortality, detailing a total of 1228 records identified (1201 excluded), 29 full-text reports screened (20 excluded), and 9 articles included. AUC: area under the curve; AUROC: area under the receiver operating curve; CML: centralized machine learning; ICU: intensive care unit; PTE: pulmonary thromboendarterectomy.

In total, 1228 records were identified, 29 full-text reports were screened, and 9 articles were included (Figure 1).

### Included Studies

Study selection was performed in 3 stages. In the first stage, 1228 studies were screened for duplication using the EndNote 20 software. In the second stage, potentially relevant studies were assessed by comparing the titles and abstracts (n=1178) against the predetermined inclusion criteria. In the third stage, studies (n=27) that appeared to meet the inclusion criteria and articles (n=2) that were sought from the citations were obtained for detailed assessment.

Among the 29 studies that were identified and assessed for eligibility, 20 were excluded because they had no comparison with CML or only included other FL-based models as the comparator group, used evaluation metrics other than the AUROC/AUC value, and were review articles or retracted articles. The characteristics and respective references of all the included studies are presented in Multimedia Appendix 3 [19-27]. Five studies were conducted in the United States, 3 in Asia, and 1 in Europe. Of the 9 studies, 8 were retrospective cohort studies using institutional data sources [19-26]. The minimum prediction window was 24 hours and the maximum was 30 days. The median sample size was 28,000 (minimum: 3055; maximum: 1,222,554). Prediction models were developed in all studies using internal validation. The most common ML techniques for the reported models were neural networks (6/9, 67%) [19-21,23-25] and logistic regression (3/9, 33%) [22,26,27].

### Risk of Bias

**Figure 2.**
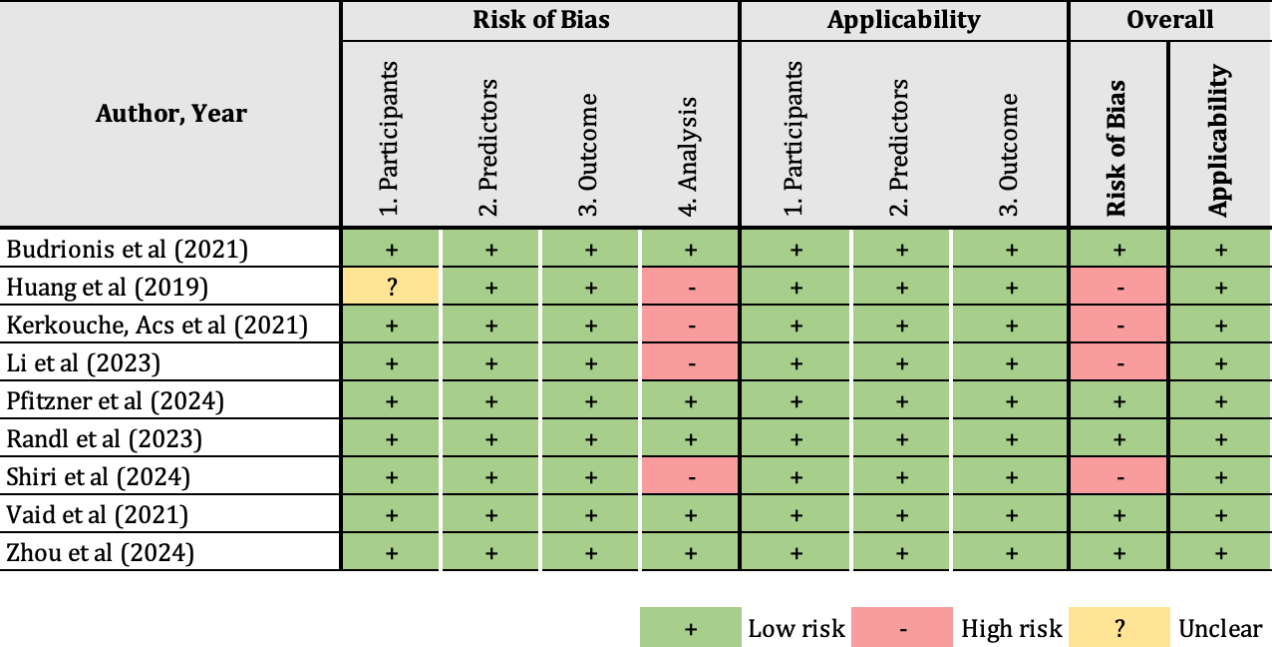
Risk of bias assessment [19-27].

In total, 5 studies were rated as having a low risk of bias [19,23,24,26,27], while 4 out of 9 of the developed models [20-22,25] were identified as having a high risk of bias (Figure 2 [19-27]; Multimedia Appendix 5 [19-27]). One study [20] did not provide information related to the preprocessing step; therefore, the data quality assessment was unclear, and this study was rated as having an unclear risk of bias in the participants’ domain (Figure 2 [19-27]). The most common reasons for the risk of bias included insufficient information regarding the number of missing data, handling of missing values, and complexities in the data. In terms of applicability, information regarding adherence to the TRIPOD (Transparent Reporting of a Multivariable Prediction Model for Individual Prognosis or Diagnosis) statement was reported in only one study [27]. However, the outcome (in this case, mortality) predicted in all developed models matched the review question. Therefore, all of the included studies could be judged as having a low risk for applicability.

### FL and Mortality Prediction: State-of-the-Art

Descriptions of the included studies are provided in Multimedia Appendix 3 [19-27]. Budrionis et al [19] developed a feed-forward neural network combined with a recurrent neural network as the baseline ML model to predict mortality among patients in ICU. Their results demonstrated that the performance of the FL model was comparable to that of the CML in terms of the *F*_1_-score and AUROC. However, the FL model training and inference required approximately 9 and 40 times longer, respectively, than the equivalent tasks that were executed in centralized settings. Using the same publicly available ICU dataset and deep learning-based approach, Randl et al [24] consistently demonstrated the comparable performance between FL and CML models in different schemes and with different numbers of clients.

Using the same ML-based model approach, logistic regression, 3 studies consistently showed that FL-based models outperformed centralized models [22,26,27]. Li et al [22] incorporated 10 simulated sites from a tertiary hospital in Singapore by implementing a scoring-based system (the FedScore model) to facilitate cross-institutional collaborations to predict mortality within 30 days after ED visits. Similarly, FL models outperformed CML in predicting the mortality of hospitalized patients with COVID-19 and pulmonary thromboendarterectomy using a real-world dataset [26,27]. Pfitzner et al [23] used a neural network-based model for predicting patient mortality and revision surgery after visceral operations, demonstrating that the FL model performed better than CML in terms of the AUROC.

Three studies showed that the centralized model still performs well when compared with FL with subtle distinctions. All 3 studies developed neural network-based prediction models. Huang et al [20] introduced a community-based FL algorithm, where distributed data were clustered into clinically meaningful communities based on similar diagnoses and geographical locations. Their evaluation showed that the community-based FL predictive performance was not substantially dissimilar from CML in predicting mortality in an ICU setting. The CML model also performed better than FL but not significantly different when predicting mortality in an emergency setting [21] and among patients with COVID-19 [25]. In the study by Shiri et al [25], the mean AUCs of 0.82 (95% CI 0.79‐0.85) and 0.81 (95% CI 0.77‐0.84) were achieved by the centralized and FL models, respectively. However, the DeLong test indicated that the differences were not statistically significant (*P*=.98).

### Predictive Performance

**Figure 3.**
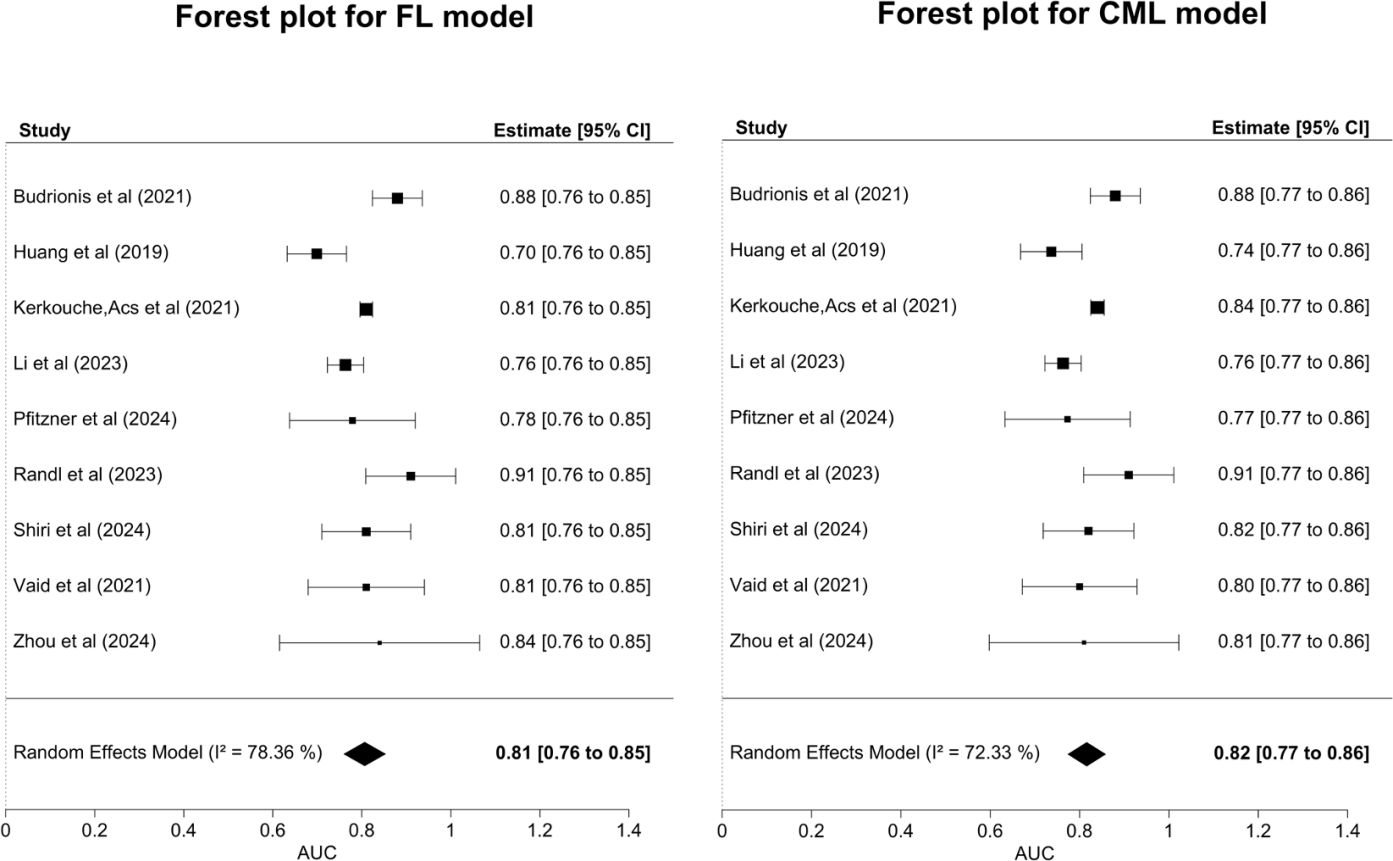
Pooled area under the curve (AUC) of federated learning (FL) and centralized machine learning (CML) [19-27].

Most studies used more than one evaluation metric to describe the performance of the developed models (eg, AUROC or AUC, sensitivity or recall, specificity, precision, accuracy, area under the precision-recall curve, and *F*_1_-score; Multimedia Appendix 3 [19-27]). The calibration performance was not reported in any of the included studies. The pooled AUC with a 95% CI and heterogeneity indices for the FL and CML performance were 0.81 (95% CI 0.76‐0.85; *I*^2^=78.36%) and 0.82 (95% CI 0.77‐0.86; *I*^2^=72.33%), respectively (Figure 3 [19-27]). The performance of FL was similar to that of CML in its ability to predict mortality in various clinical settings.

## Discussion

### Principal Results

This study reveals the current state of research related to the implementation of the FL approach in health care. More than half (5/9, 56%) of the studies were found in medical journals, which suggests that the clinical use of FL in the medical domain is increasingly gaining popularity. Compared with the nonprivate CML, the FL-based models exhibited sufficient discrimination ability in predicting mortality when operated across various clinical settings (ie, ICU, ED, and specific disease). There is evidence that FL-based models performed similarly [19,24] to or better than CML [22,23,26,27] when developed using different predictors (eg, laboratory values, vital signs) in distinct schemes (ie, configuration setting, scoring-based) and prediction windows (ie, 24 h, 48 h, 72 h, 96 h, 7 days, and 30 days). This performance persists despite the given cost and complexity associated with node orchestration, privacy preservation, and extra steps that do not exist in centralized approaches. The pooled AUC values for FL and CML were 0.81 (95% CI 0.76‐0.85) and 0.82 (95% CI 0.77‐0.86), respectively, proving the feasibility of FL implementation in a health care setting given the high imbalance and nonindependent and identically distributed (non-IID) nature of the clinical dataset.

All of the included studies reported findings in favor of the FL approach. The performance of the FL model was not affected by the number of computational nodes or data distribution across the nodes, which concurred with the existing literature on the implementation of FL [13]. However, there was a considerable complication effect of FL algorithms in terms of the training time, inference duration, and communication rounds. The training and prediction times of the FL model were reported to be 9 and 40 times longer than those of the CML model, respectively [19]. This finding is supported by a prior study that revealed the effect of FL infrastructure computational overheads in increasing the inference time duration [4,12,13]. Although the inference duration seems relatively high compared with that of CML, the previous work suggested that in real-life deployments, predictions are likely to be made for individual patients rather than large patient groups. Therefore, longer times for making predictions are likely negligible [28]. The communication rounds, which indicated the learning speed of the model, were reported to be 57 times slower in FL than in CML [20]; that is, the FL algorithm required higher communication costs between the hospital and server to achieve convergence. The iterative nature of the FL model, in which each round must ensure that all relevant updates are incorporated into the global model for convergence, has been explored extensively in previous experimental studies. However, it has been suggested that the robustness of the FL performance was not affected [4,12,29,30].

Although no study has quantitatively summarized the discriminant ability of FL compared with CML in clinical settings, it has been qualitatively demonstrated that FL models enhance the generalizability and analysis power while conquering privacy risks [4,12,13]. Consistent with this finding, our meta-analysis found insubstantial differences in the pooled AUC between the FL and CML for predicting mortality in various scenarios. Similar to a previous systematic review [13], we found that most of the developed FL models used a neural network approach, whereas the remaining minority used logistic regression. This may correlate with the data type used in the current FL research, where neural networks have been shown to provide excellent performance. In addition, the large variety of data types, both structured and unstructured, that have been successfully used in FL models is encouraging.

The FL model was not only experimentally trained using supervised data type. The use of radiological data was reported by Shiri et al [25]. In the field of medical imaging, data annotation is one of the crucial and labor-intensive tasks. Through the incorporation of the FL-based model, different institutions can benefit from each other’s annotations without even sharing them. Training deep learning algorithms requires high computational power and memory space. The use of the FL model offers the promise to enhance efficiency in training and memory consumption for AI-assisted medical image analysis algorithms [31].

Previous studies have reported that the global FL model is more robust and achieves greater accuracy at individual sites than models trained solely on local data for predicting mortality [22,23,27]. This improvement is likely attributable to the availability of larger and more diverse datasets, the use of input data that can be standardized, and the avoidance of clinical impressions or reported symptoms. These factors collectively enhance the benefits of the FL approach and its impact on performance, generalizability, and ultimately, the model’s usability in the clinical domain. For client sites with relatively small datasets, 2 common approaches could be used for fitting a useful model: one is to train locally with its own data, and the other is to apply a model trained on a larger dataset [32]. The finding is that the global FL model can increase the accuracy of the locally trained model to predict mortality in ED and non-ED admissions while the number of patients was relatively small with a low percentage of desirable outcomes [22,23,27], indicating that the benefit for client sites with small datasets arising from participation in FL collaborations is substantial.

Consistent with the prior study, the FL model was proved to have the ability to capture more diversity than local training and to mitigate the bias present in models trained on a homogenous population [32]. In clinical domains, however, data are frequently formed at the hospital or institution level, making local models feasible in these cases. Under these circumstances, the generalizability and stability of global models relative to local models become more crucial. Li et al [22] showed that by a cotraining process via FL, a global model prediction framework such as FedScore can achieve less variation than locally developed ones while still maintaining good performance. This benefit of FL is promising for medical research that seeks dependable high-risk decision-making.

Although FL mitigates privacy risks by design, certain attacks, such as membership and property inference attacks, are still possible [21,33]. However, in line with previous studies [34-37], we found that implementing differential privacy (DP) to mitigate inference attacks in the FL model remains challenging. Although DP adds an extra layer of privacy protection, a trade-off exists between privacy, accuracy, and model fairness in FL with DP [35]. We found that strong privacy protection can be provided at the cost of performance degradation [21,23,25].

FL faces significant challenges due to data heterogeneity, which refers to the nonuniform distribution of data across participating clients. This heterogeneity arises from differences in data types, feature distributions, and class imbalances. We found that both evenly distributed and non-IID datasets used for developing FL-based models were reported. A previous study found that non-IID data can significantly reduce the model accuracy, which is explained by the weight divergence between local and global distributions [38,39]. Consistent with the existing literature [38,40], the use of hierarchical local clustering to improve convergence and accuracy was also proposed by our findings [20]. A previous study reported that clustering patients with common features into the same community and learning separate models for individual communities not only enhances the predictive accuracy in fewer communication rounds but also allows for the interpretability of the prediction results [40].

In line with the previous literature [41,42], most implementations of the prediction model development were performed using a retrospective cohort, extracted from a publicly available dataset, rather than a clinical study design. According to our findings, most of the included studies were in the development phase, in which the models were tested and optimized without external validation. Thus, significant development is still required to improve the maturity of technologies during the conceptualization, development, and application stages. In addition, the models must be tested using real-time data. Additional development is also necessary to introduce the models to the clinical workflow, evaluate clinical outcomes, and integrate the models into the hospital environment.

The included studies demonstrated sufficient discrimination ability, which is a prerequisite for clinical acceptance [41,42]. However, prior to this, external validation within a clinical workflow must be established. In future research, it will be crucial to ascertain whether the model encompasses both treated and untreated patients and how the treatment effects are handled in the models. Furthermore, the establishment of a real-time data infrastructure is imperative for effectively coping with unknowns.

Offering global collaboration, predictive power, and privacy preservation through FL, medical institutions worldwide could share insights and collectively train predictive models for treatment strategies while safeguarding patient information. FL’s decentralized nature allowed for real-time data analysis and rapid response. Health care professionals and researchers could continuously update and refine models as new data became available, leading to more accurate predictions and recommendations. Additionally, FL facilitated global collaboration, enabling experts from various regions to pool their data effectively. This collaborative approach is instrumental in improving care strategies, as researchers worldwide could collectively analyze clinical trial data without compromising data privacy. Overall, FL emerged as a crucial tool that enables privacy-preserving data collaboration, real-time analysis, and global cooperation among health care professionals and researchers.

### Strengths and Limitations

The key strengths of this systematic review are the quantitative meta-analytical methods that allowed for robust conclusions based on cumulative evidence regarding the feasibility of FL approaches for clinical implementation. Focusing on the pooled AUC, which is a well-established metric for evaluating the discriminatory ability of predictive models, this study provides a clear and quantifiable measure of how well each model predicts mortality risk, thereby facilitating straightforward comparisons. By aggregating data from multiple studies in different clinical settings, this meta-analysis has captured a wide range of clinical environments and patient populations to provide clinicians with reliable information on which predictive modeling approach may be more effective in their specific settings. In addition, this study offers more precise estimates of the model performance to aid in identifying subtle differences between the FL and CML approaches owing to the involvement of over a million study participants.

This study has several limitations. First, gray literature was not included. In addition, meta-regression was not conducted because of the small number of studies. Second, the high risk of bias most often originated in the analysis domain owing to values not being reported or the inappropriate handling of missing values, as well as methods for dealing with data complexities not being reported. In addition, differences in the predictors, prediction windows, study characteristics, and clinical settings were potential sources of heterogeneity among the included studies. Moreover, these studies included only adult patients. This may pose challenges to generalizability and fairness when applied to a broader population. Finally, the calibration performance was not evaluated because of the lack of studies. The lack of calibration in FL models can severely limit their applicability across different populations.

### Conclusions

In conclusion, FL-based models can achieve a performance similar to that of centralized models trained on pooled data while preserving data privacy in predicting mortality across various clinical settings. This study demonstrates the feasibility of using FL models to construct a risk prediction model for mortality prediction while addressing data privacy concerns, which is helpful for clinical practice. However, the included studies only performed an internal validation of the data, and researchers should be encouraged to perform and report external validation of the available models. The former type of studies often overestimated the true predictive performance. Future research directions include a repetition of this review to keep up with the rapidly growing use of FL-based models in the clinical environment, and further evaluation and exploration of how FL is performed in different groups of patients and specific cases to assess research evidence.

## Supplementary material

10.2196/65708 Multimedia Appendix 1 Search strategy.

10.2196/65708 Multimedia Appendix 2 Prediction model study Risk Of Bias Assessment Tool (PROBAST) Signaling Question.

10.2196/65708 Multimedia Appendix 3 Description of the included articles and full details of meta-analyses.

10.2196/65708 Multimedia Appendix 4 Model summary and approximation formula.

10.2196/65708 Multimedia Appendix 5 Risk of bias assessment by CHARMS (Checklist for Critical Appraisal and Data Extraction for Systematic Reviews of Prediction Modeling Studies) and PROBAST (Prediction model study Risk Of Bias Assessment Tool) guidelines.

10.2196/65708 Checklist 1 PRISMA (Preferred Reporting Items for Systematic Reviews and Meta-Analyses) checklist.

## Abbreviations

AUC: area under the curve
AUROC: area under the receiver operating curve
CHARMS: Checklist for Critical Appraisal and Data Extraction for Systematic Reviews of Prediction Modeling Studies
CML: centralized machine learning
DP: differential privacy
ED: emergency department
ED: emergency department
FL: federated learning
ICU: intensive care unit
ML: machine learning
PRISMA: Preferred Reporting Items for Systematic Reviews and Meta-Analyses
PROBAST: Prediction Model Risk of Bias Assessment Tool
TRIPOD: Transparent Reporting of a Multivariable Prediction Model for Individual Prognosis or Diagnosis
