# Supplementary material for: Federated Learning-Based Model for Predicting Mortality: Systematic Review and Meta-Analysis
Source: J Med Internet Res. 2025 Jul 21;27:e65708. doi: 10.2196/65708 (PMC12303363; doi:10.2196/65708)
Supplement: Multimedia Appendix 1 [file jmir-v27-e65708-s001.docx]

Multimedia Appendix 1

Search strategy

| **Databases** | **Search terms** |
| --- | --- |
| **IEEE Xplore** | ("federated learning" OR "federated machine learning") AND (mortality OR clinical OR "clinical outcome") AND (prediction) |
| **PubMed** | ("federated learning" OR "federated machine learning") AND (mortality OR clinical OR "clinical outcome") AND (prediction) |
| **Science Direct** | ("federated learning" OR "federated machine learning") AND (mortality OR clinical OR "clinical outcome") AND (prediction) |
| **Web of Science** | ("federated learning" OR FL OR federated OR "decentralized learning") AND (mortality) AND (prediction OR detection OR "early detection") |
