# Supplementary material for: Federated Learning-Based Model for Predicting Mortality: Systematic Review and Meta-Analysis
Source: J Med Internet Res. 2025 Jul 21;27:e65708. doi: 10.2196/65708 (PMC12303363; doi:10.2196/65708)
Supplement: Multimedia Appendix 2 [file jmir-v27-e65708-s002.docx]

Multimedia Appendix 2

Prediction model study Risk Of Bias Assessment Tool (PROBAST) Signaling Question

| **DOMAIN 1: Participants** |
| --- |
| - 1. Were appropriate data sources used? |
| - 1. Were all inclusions and exclusions of participants appropriate? |
| **DOMAIN 2: Predictors** |
| 2.1 Were predictors defined and assessed in a similar way for all participants? |
| 2.2 Were predictor assessments made without knowledge of outcome data? |
| 2.3 Are all predictors available at the time the model is intended to be used? |
| **DOMAIN 3: Outcome** |
| 3.1 Was the outcome determined appropriately? |
| 3.2 Was a pre-specified or standard outcome definition used? |
| 3.3 Were predictors excluded from the outcome definition? |
| 3.4 Was the outcome defined and determined in a similar way for all participants? |
| 3.5 Was the outcome determined without knowledge of predictor information? |
| 3.6 Was the time interval between predictor assessment and outcome determination appropriate? |
| **DOMAIN 4: Analysis** |
| 4.1 Were there a reasonable number of participants with the outcome? |
| 4.2 Were continuous and categorical predictors handled appropriately? |
| 4.3 Were all enrolled participants included in the analysis? |
| 4.4 Were participants with missing data handled appropriately? |
| 4.5 Was selection of predictors based on univariable analysis avoided? |
| 4.6 Were complexities in the data accounted for appropriately? |
| 4.7 Were relevant model performance measures evaluated appropriately? |
| 4.8 Were model overfitting and optimism in model performance accounted for? |
| 4.9 Do predictors and their assigned weights in the final model correspond to the results from multivariable analysis? |
