# Supplementary material for: Federated Learning-Based Model for Predicting Mortality: Systematic Review and Meta-Analysis
Source: J Med Internet Res. 2025 Jul 21;27:e65708. doi: 10.2196/65708 (PMC12303363; doi:10.2196/65708)
Supplement: Multimedia Appendix 3 [file jmir-v27-e65708-s003.docx]

Multimedia Appendix 3

Description of the included articles and full details of meta-analyses

| Author(s),  year, ref no | Cohort name, country, follow-up duration | Number of participants | Inclusion (**I**)/ Exclusion (**E**) Criteria | Type of prediction model study (Development/Validation) | Outcome to be predicted (Prognostic/Diagnostic) | Predictors | Model development | Comparator (**C**) and Intervention Approach (**IA**) | Evaluation Metric | Result (best performance of comparator vs intervention approach) |
| --- | --- | --- | --- | --- | --- | --- | --- | --- | --- | --- |
| Budrionis et al (2021) [1] | MIMIC-III, United State, 7 days | 35,627 | **I**: all the patients whose age was>15 years at the time of ICU admission, first ICU admission for each patient **E**: patient later ICU admission | Development | Prognostic | 17 features used in SAPS-II scoring system | Feed-forward networks (FFN) with sigmoid output layer | **C**: CML **IA**: FL in different configuration of data, nodes, and distribution | F1-Score, ROC AUC, training and inference durations (seconds) | **Data configuration** **F1-Score**:  CML= 0.27 – 0.37 FL= 0.05 – 0.30 **ROC AUC** CML= 0.67-0.88 FL = 0.60-0.88 **Training time** CML= 0-9 (s) FL = 0-700 (s) **Inference duration** CML= 0 (s) FL = 12 (s) |
| Huang et al (2019) [2] | e-ICU Collaborative Research Database by Philips Healthcare, United State, NI | 28,000 | **I**: patients with drugs administered during the first 48h of ICU stay **E**: - | Development | Prognostic | Drugs administered on patients during the first 48h of ICU stay (n= 1399) | Community-based federated machine learning (CBFL) | **C**: CML **IA**: FL and CBFL in two different setting of data set (using the same source for training and testing data set; using different sources for training and testing dataset) | ROC AUC, PR AUC, and communication rounds | **Training and testing data set come from the same source** **ROC AUC** CML = 0.7368 FL = 0.6895 CBFL10 = 0.6989 **PR AUC** CML = 0.1449 FL = 0.1107 CBFL10 = 0.1070 **Communication round** CML = - FL = 101 CBFL10 = 57 |
| Kerkouche et al (2021) [3] | Premier healthcare database, United State, 24h | 1,222,554 | **I**: adult hospitalized patients whose had recorded for at least 3 days **E**: patient with elective admission, pregnant and new-born patients | Development | Prognostic | Age, gender, admission type, MRCI, Drugs and ICD9 codes (n = 24,428) | FL-SIGN-DP: each client sends only the sign of every coordinate value in its parameter update vector with differential privacy | C: CML IA: FL in different approaches (FL-STANDARD, FL-SIGN, FL-STANDARD-DP, FL-SIGN-DP) | AUROC, balanced accuracy | **AUROC** CML = (0.82,0.84)  FL-SIGN = (0.76,0.77) FL-STANDARD = (0.79,0.81) **Balanced accuracy** CML = (0.76,0.77)  FL-SIGN = (0.68,0.70)  FL-STANDARD = (0.73,0.74) |
| Li et al (2023) [4] | Singapore General Hospital (SGH) extracted from the SingHealth Electronic Health Intelligence System, Singapore, 30 days | 80,613 | **I**: patients with emergency department (ED) visits **E**: patients under the age of 18 and those with missing values | Development | Prognostic | Demographics information, PACS triage categories, vital signs, comorbidities, and previous health care usage (n = 29) | FedScore (federated learning framework for scoring system generation) | **C**: CML and LML **IA**: FL with scoring system | Mean and SD of AUC | **Mean of AUC** CML = 0.7631 FL = 0.7633  **SD** CML = 0.0289 FL = 0.0204 |
| Pfitzner et al (2024) [5] | Department of Surgery, Campus Charit´ e Mitte, Campus Virchow Klinikum, Charit´ e– Universitatsmedizin Berlin, NI | 6,774 | **I**: received elective or urgent major surgery of either the colorectum, liver, oesophagus, pancreas or stomach, over the age of 18 **E**: - | Development | Prognostic | 60 preoperative features | Federated neural network | C: CML and LML (with and without DP) IA: FL with and without DP | AUPRC, F1-Score, AUROC | **Without DP** **AUPRC** CML = 0.523±0.036 FL = 0.514±0.053 **F1-Score**  CML = 0.517±0.024 FL = 0.504±0.041 **AUROC**  CML = 0.755±0.018 FL = 0.743±0.036 |
| Randl et al (2023) [6] | MIMIC-III, United State, 24h, 48h, 72h, 96h | 28,324 | **I**: patients with the first ICU stay **E**: patients with data recorded for < Δtmin, patients staying longer than Δtmax, patients from the Neonatal Intensive Care Unit (NICU), and Pediatric Intensive Care Unit (PICU) | Development | Prognostic | demographic information, vital signs, lab values (n = 25) | Deep federated learning with less resource -intensive Gated Recurrent Unit (GRU) | **C**: CML and LML **IA**: FL in two schemes (using early stop with minimum loss and early stop with maximum F1-Score) and different amounts of clients (2, 4, and 8) | AUROC, AUPRC, F1-Score, precision, recall | **Early stop with maximum F1-Score (2 clients) AUROC** CML = 0.90±0.01 FL = 0.90±0.01 **AUPRC**  CML = 0.50±0.04 FL = 0.49±0.04 **F1-Score**  CML = 0.49±0.04 FL = 0.48±0.03 **Precision**  CML = 0.55±0.05 FL = 0.52±0.05 **Recall**  CML = 0.46±0.08 FL = 0.46±0.05 |
| Shiri et al (2024) [7] | 19 centers in Iran, NI | 3,055 | **I**: patients’ chest CT images, RT-PCR results, and the results of a 4-month follow-up for outpatient centers or at-discharge follow-up for inpatient ones based on which the subjects were sub-grouped into “alive” and “deceased” **E**: patients not followed up or transferred to another hospital, cases with a confirmed diagnosis of lung cancer at any stage, subjects whose CT images sustained motion artifacts or incomplete lung presentation or low quality, patients who had negative RT-PCR, and cases with only contrast-enhanced CT images | Development | Prognostic | Patient demographics and CT acquisition parameters (n = 4) | Gaussian differentially private federated weighted averaging with an adaptive quantile clipping (GDP-AQuCl) | **C**: CML **IA**: FL | Precision, sensitivity, specificity, F1, accuracy, balanced accuracy, FNR, FDR, FPR, NPV, PPV, AUC | **Precision** CML = 0.74 FL = 0.73 **Sensitivity** CML = 0.74 FL = 0.73  **Specificity** CML = 0.77 FL = 0.77  **F1** CML = 0.74 FL = 0.73  **Accuracy** CML = 0.76 FL = 0.75  **Balanced accuracy** CML = 0.76 FL = 0.75  **FNR** CML = 0.26 FL = 0.27  **FDR** CML = 0.26 FL = 0.27  **FPR** CML = 0.23 FL = 0.23  **NPV** CML = 0.78 FL = 0.76  **PPV** CML = 0.74 FL = 0.73  **AUC** CML = 0.82 FL = 0.81 |
| Vaid et al (2021) [8] | 5 hospitals within the Mount Sinai Health System, United State, 7 days | 4,029 | **I**: Hospitalized patients at 5 NYC hospitals in the Mount Sinai Health System, patients >18 years of age with a positive SARS-CoV2 RT-PCR test that was placed within 48 hours of admission and were intubated <48 hours after admission **E**: - | Development | Prognostic | Demographics, medical history, vitals at intake, and labs on admission (within 36h) (n = 68) | Federated Multilayer Perceptron (MLP) and L1 regularization/least absolute shrinkage and selection operator (LASSO) model | **C**: CML and LML **IA**: FL developed in two baseline model, MLP and LASSO model | AUROC | **MLP Model Average of AUROC from 5 sites** CML = 0.80  FL = 0.81 |
| Zhou et al (2024) [9] | 12 hospitals in China, 30 days | 3,997 | **I**: inpatients aged 18 or older who were diagnosed with PTE; only the first available medical record was included for patients with repeated hospitalizations due to PTE **E**: patients who were diagnosed and transferred from other healthcare facilities due to incomplete medical records | Development | Prognostic | Demographic information, medical history, related risk factors, and 30-day PTE all-cause mortality (n = 19) | Logistic Regression (LR) | **C**: CML and LML **IA**: FL using real world data, under IID, and non-IID data | AUC, AUPRC, accuracy, precision, recall, F1-Score, | **Real world data AUC** CML = 0.811 ± 0.001 FL = 0.835 ± 0.005 **AUPRC** CML = 0.291 ± 0.002 FL = 0.311 ± 0.011  **Accuracy** CML = 0.810 ± 0.040 FL = 0.970 ± 0.002  **Precision** CML = 0.109 ± 0.014 FL = 0.792 ± 0.158  **Recall** CML = 0.606 ± 0.092 FL = 0.176 ± 0.028  **F1-Score** CML = 0.183 ± 0.014 FL = 0.287 ± 0.040 |

**References**

1. Budrionis A, et al. Benchmarking PySyft federated learning framework on MIMIC-III dataset. IEEE Access. 2021;9:116869-116878.
2. Huang L, Shea AL, Qian H, Masurkar A, Deng H, Liu D. Patient clustering improves efficiency of federated machine learning to predict mortality and hospital stay time using distributed electronic medical records. J Biomed Inform. Nov 2019;99:103291. [doi: 10.1016/j.jbi.2019.103291] [Medline: 31560949]
3. Kerkouche R, et al. Privacy-preserving and bandwidth-efficient federated learning: an application to in-hospital mortality prediction. Presented at: Proceedings of the Conference on Health, Inference, and Learning Virtual Event, USA, Association for Computing Machinery. 25-35. 2021.
4. Li S, Ning Y, Ong MEH, et al. FedScore: a privacy-preserving framework for federated scoring system development. J Biomed Inform. Oct 2023;146:104485. [doi: 10.1016/j.jbi.2023.104485] [Medline: 37660960]
5. Pfitzner B, Maurer MM, Winter A, et al. Differentially-private federated learning with non-IID data for surgical risk prediction. Presented at: 2024 IEEE First International Conference on Artificial Intelligence for Medicine, Health and Care (AIMHC). 2024.[doi: 10.1109/AIMHC59811.2024.00030]
6. Randl K, Lladós Armengol N, Mondrejevski L, Miliou I. Early prediction of the risk of ICU mortality with deep federated learning. Presented at: 2023 IEEE 36th International Symposium on Computer-Based Medical Systems (CBMS); L’Aquila, Italy. 2023.[doi: 10.1109/CBMS58004.2023.00304]
7. Shiri I, Salimi Y, Sirjani N, et al. Differential privacy preserved federated learning for prognostic modeling in COVID-19 patients using large multi-institutional chest CT dataset. Med Phys. Jul 2024;51(7):4736-4747. [doi: 10.1002/mp.16964] [Medline: 38335175]
8. Vaid A, Jaladanki SK, Xu J, et al. Federated learning of electronic health records to improve mortality prediction in hospitalized patients with COVID-19: machine learning approach. JMIR Med Inform. Jan 27, 2021;9(1):e24207. [doi: 10.2196/24207] [Medline: 33400679]
9. Zhou J, Wang X, Li Y, Yang Y, Shi J. Federated-learning-based prognosis assessment model for acute pulmonary thromboembolism. BMC Med Inform Decis Mak. May 27, 2024;24(1):141. [doi: 10.1186/s12911-024-02543-x] [Medline: 38802861]
