# Supplementary material for: Federated Learning-Based Model for Predicting Mortality: Systematic Review and Meta-Analysis
Source: J Med Internet Res. 2025 Jul 21;27:e65708. doi: 10.2196/65708 (PMC12303363; doi:10.2196/65708)
Supplement: Multimedia Appendix 4 [file jmir-v27-e65708-s004.docx]

Multimedia Appendix 4

Model summary and approximation formula

Variance Estimator

When the within-study variance of the c-statistic, Var(c), is not known it is still possible to approximate the within-study variance of the logit c-statistic [1].

$Var(logit(c))$ ≈ $\left( \frac{\partial logit\left( c \right)}{\partial c} \right)^{2} Var(c)$

≈ $\frac{Var(c)}{(c\left( 1-c \right))^{2}}$

≈ $\frac{1 +(N/2 - 1)(1-c)/(2-c)+(N/2 - 1)c/(1+c)}{c\left( 1-c \right)O(N-0)}$

where *N* is the total sample size and *O* is the number of observed events

Summary of FL Model


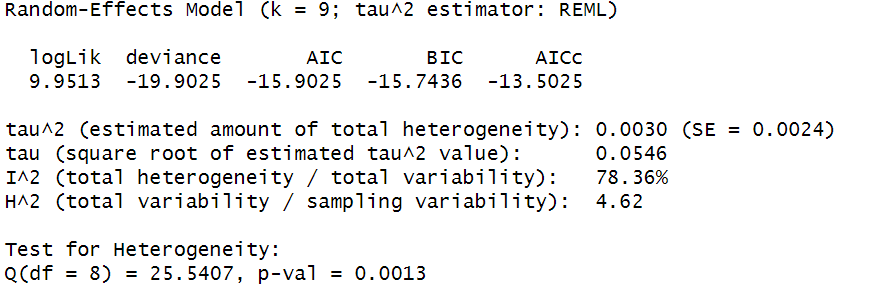


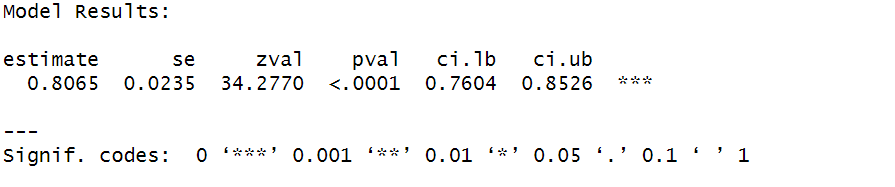


Summary of CML model


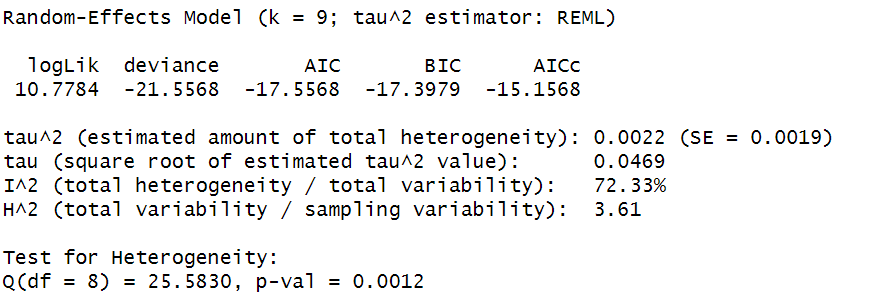


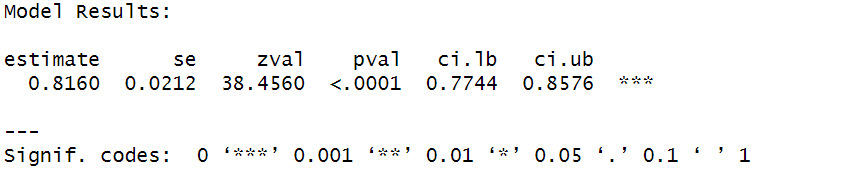


Summary of FL Model for Subgroup


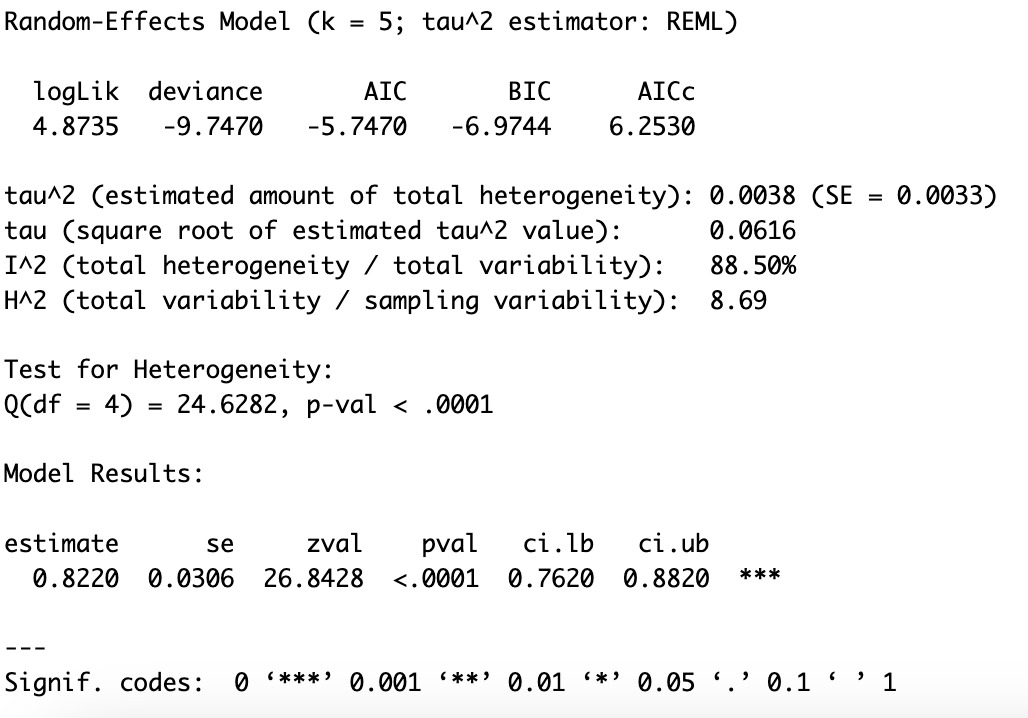


Summary of CML Model for Subgroup


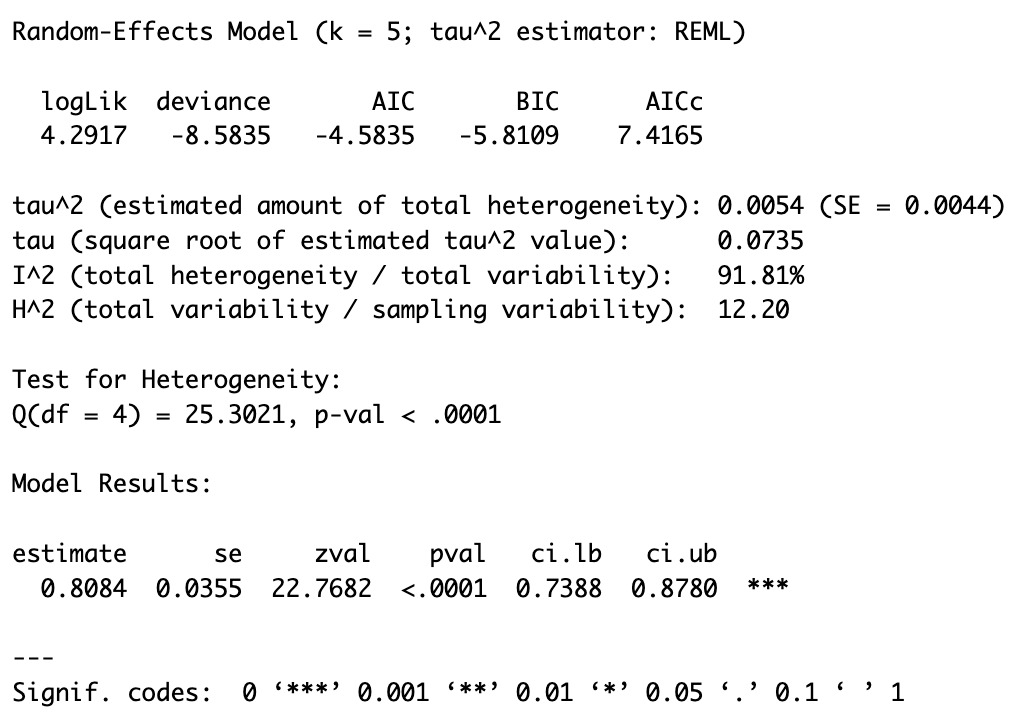


**Reference**

1. Randl K, Lladós Armengol N, Mondrejevski L, Miliou I. Early prediction of the risk of ICU mortality with deep federated learning. Presented at: 2023 IEEE 36th International Symposium on Computer-Based Medical Systems (CBMS); L’Aquila, Italy. 2023.[doi: 10.1109/CBMS58004.2023.00304]
