# Supplementary material for: Federated Learning-Based Model for Predicting Mortality: Systematic Review and Meta-Analysis
Source: J Med Internet Res. 2025 Jul 21;27:e65708. doi: 10.2196/65708 (PMC12303363; doi:10.2196/65708)
Supplement: Multimedia Appendix 5 [file jmir-v27-e65708-s005.docx]

Multimedia Appendix 5

Risk of bias assessment by CHARMS and PROBAST guideline

| **Author, Year** | **Modelling method** | **Sample size** | **Events** | **No predictors** | | **EPV or EPP** | **Selection of candidate predictors** | **Selection of final predictors** | **Number (%) and handling of missing data** | **Type of validation** | **Performance measures** |  | **Critical appraisal (PROBAST)** | | | |
| --- | --- | --- | --- | --- | --- | --- | --- | --- | --- | --- | --- | --- | --- | --- | --- | --- |
|  |  |  | **n (%)** | **Cand.** | **Final** |  |  |  |  |  |  |  | **P** | **Pr** | **O** | **A** |
| Budrionis et al (2021) [1] | Neural network | 35,627 | 3738 (10.5) | 17 | 17 | 219.9 | Based on prior knowledge | Other | n (%): Unknown Method: Multiple imputation | Int: Random split data Ext: None | Cal: Not evaluated Disc: C-Statistic / AUC graph  Ov: F1-Score and ROC AUC | **RoB** | + | + | + | + |
|  |  |  |  |  |  |  |  |  |  |  |  | **App** | + | + | + |  |
| Huang et al (2019) [2] | Neural network | 28,000 | 1395 (5.0) | 1399 | 1399 | 1.0 | Based on prior knowledge | Other | n (%): Unknown Method: No information | Int: Random split data Ext: None | Cal: Not evaluated Disc: C-Statistic / AUC graph  Ov: ROC AUC and PR AUC | **RoB** | ? | + | + | - |
|  |  |  |  |  |  |  |  |  |  |  |  | **App** | + | + | + |  |
| Kerkouche et al (2021) [3] | Neural network | 1,222,554 | 38,666 (3.2) | 24,428 | 24,428 | 1.6 | Based on prior knowledge | No information | n (%): Unknown Method: No information | Int: Random split data Ext: None | Cal: Not evaluated Disc: C-Statistic / AUC graph  Ov: AUROC | **RoB** | + | + | + | - |
|  |  |  |  |  |  |  |  |  |  |  |  | **App** | + | + | + |  |
| Li et al (2023) [4] | Machine learning techniques | 80,613 | 4249 (5.3) | 29 | 29 | 146.5 | Based on prior knowledge | Other | n (%): Unknown Method: Complete-case analysis | Int: Random split data Ext: None | Cal: Not evaluated Disc: C-Statistic / AUC graph  Ov: Mean and SD of AUC | **RoB** | + | + | + | - |
|  |  |  |  |  |  |  |  |  |  |  |  | **App** | + | + | + |  |
| Pfitzner et al (2024) [5] | Neural network | 6,774 | 366 (5.4) | 60 | 60 | 6.1 | Based on prior knowledge | No information | n (%): Unknown Method: Single imputation | Int: Random split data Ext: None | Cal: Not evaluated Disc: AUC graph  Ov: AUPRC, F1-Score, AUROC | **RoB** | + | + | + | + |
|  |  |  |  |  |  |  |  |  |  |  |  | **App** | + | + | + |  |
| Randl, et al. (2023) [6] | Neural network | 28,324 | 1351 (4.8) | 25 | 25 | 54.0 | Based on prior knowledge | Other | n (%): Unknown Method: Multiple imputation | Int: Random split data Ext: None | Cal: Not evaluated Disc: AUC graph  Ov: AUROC, AUPRC, F1-Score, precision, recall | **RoB** | + | + | + | + |
|  |  |  |  |  |  |  |  |  |  |  |  | **App** | + | + | + |  |
| Shiri et al (2024) [7] | Neural network | 3,055 | 1456 (47.7) | 4 | 4 | 364.0 | Based on prior knowledge | Ridge regression | n (%): Unknown Method: No information | Int: Random split data Ext: None | Cal: Not evaluated Disc: C-Statistic / AUC graph  Ov: Precision, sensitivity, specificity, F1, accuracy, balanced accuracy, FNR, FDR, FPR, NPV, PPV, AUC | **RoB** | + | + | + | - |
|  |  |  |  |  |  |  |  |  |  |  |  | **App** | + | + | + |  |
| Vaid et al (2021) [8] | Machine learning techniques | 4,029 | 510 (12.7) | 68 | 68 | 7.5 | Based on prior knowledge | LASSO selection | n (%): Unknown Method: Single imputation | Int: Bootstrap Ext: None | Cal: Not evaluated Disc: C-Statistic / AUC graph  Ov: AUROC | **RoB** | + | + | + | + |
|  |  |  |  |  |  |  |  |  |  |  |  | **App** | + | + | + |  |
| Zhou et al (2024) [9] | Logistic regression | 3,997 | 176 (4.4) | 19 | 19 | 9.3 | Based on prior knowledge | No information | n (%): Unknown Method: Single imputation | Int: Random split data Ext: None | Cal: Not evaluated Disc: C-Statistic / AUC graph  Ov: AUC, AUPRC, accuracy, precision, recall, F1-Score | **RoB** | + | + | + | + |
|  |  |  |  |  |  |  |  |  |  |  |  | **App** | + | + | + |  |

**References**

1. Budrionis A, et al. Benchmarking PySyft federated learning framework on MIMIC-III dataset. IEEE Access. 2021;9:116869-116878.
2. Huang L, Shea AL, Qian H, Masurkar A, Deng H, Liu D. Patient clustering improves efficiency of federated machine learning to predict mortality and hospital stay time using distributed electronic medical records. J Biomed Inform. Nov 2019;99:103291. [doi: 10.1016/j.jbi.2019.103291] [Medline: 31560949]
3. Kerkouche R, et al. Privacy-preserving and bandwidth-efficient federated learning: an application to in-hospital mortality prediction. Presented at: Proceedings of the Conference on Health, Inference, and Learning Virtual Event, USA, Association for Computing Machinery. 25-35. 2021.
4. Li S, Ning Y, Ong MEH, et al. FedScore: a privacy-preserving framework for federated scoring system development. J Biomed Inform. Oct 2023;146:104485. [doi: 10.1016/j.jbi.2023.104485] [Medline: 37660960]
5. Pfitzner B, Maurer MM, Winter A, et al. Differentially-private federated learning with non-IID data for surgical risk prediction. Presented at: 2024 IEEE First International Conference on Artificial Intelligence for Medicine, Health and Care (AIMHC). 2024.[doi: 10.1109/AIMHC59811.2024.00030]
6. Randl K, Lladós Armengol N, Mondrejevski L, Miliou I. Early prediction of the risk of ICU mortality with deep federated learning. Presented at: 2023 IEEE 36th International Symposium on Computer-Based Medical Systems (CBMS); L’Aquila, Italy. 2023.[doi: 10.1109/CBMS58004.2023.00304]
7. Shiri I, Salimi Y, Sirjani N, et al. Differential privacy preserved federated learning for prognostic modeling in COVID-19 patients using large multi-institutional chest CT dataset. Med Phys. Jul 2024;51(7):4736-4747. [doi: 10.1002/mp.16964] [Medline: 38335175]
8. Vaid A, Jaladanki SK, Xu J, et al. Federated learning of electronic health records to improve mortality prediction in hospitalized patients with COVID-19: machine learning approach. JMIR Med Inform. Jan 27, 2021;9(1):e24207. [doi: 10.2196/24207] [Medline: 33400679]
9. Zhou J, Wang X, Li Y, Yang Y, Shi J. Federated-learning-based prognosis assessment model for acute pulmonary thromboembolism. BMC Med Inform Decis Mak. May 27, 2024;24(1):141. [doi: 10.1186/s12911-024-02543-x] [Medline: 38802861]
